# Early Risk Stratification and Mortality Prediction in Gastrointestinal Perforation: A Retrospective Cohort Study for Personalized Surgical Decision Making

**DOI:** 10.3390/jpm16060289

**Published:** 2026-05-27

**Authors:** Giulia Colombo, Marco Longhi, Matteo Capuzzo, Pietro Bisagni

**Affiliations:** 1 Department of General Surgery, ASST Lodi—Ospedale Maggiore di Lodi, 26900 Lodi, Italy; marco.longhi@asst-lodi.it (M.L.); matteo.capuzzo@asst-lodi.it (M.C.); 2 General Surgery Department, Aziende Socio Sanitarie Territoriale Lodi, 26900 Lodi, Italy; 3 General Surgery Department, University of Milan, 20122 Milan, Italy

**Keywords:** gastrointestinal perforation, emergency surgery, septic shock, vasopressors, mortality, risk stratification, personalized medicine, frailty, logistic regression, surgical futility

## Abstract

**Background:** Gastrointestinal perforation is a life-threatening surgical emergency associated with high morbidity and mortality despite advances in imaging, perioperative care, and emergency surgical management. Early identification of patients at increased risk of death may improve perioperative risk stratification and support personalized clinical decision-making in emergency settings. **Methods:** We conducted a retrospective observational cohort study including 166 consecutive adult patients undergoing emergency surgery for gastric, duodenal, ileal, colonic, or intraperitoneal rectal perforation between January 2021 and December 2025. Appendiceal perforations, iatrogenic perforations, and anastomotic leaks were excluded. Univariate analysis was performed using appropriate non-parametric and categorical statistical tests. Variables with *p* < 0.05 were considered for multivariable logistic regression analysis. Postoperative variables potentially influenced by the outcome were excluded to reduce reverse causation and overadjustment bias. Age was analyzed as a continuous variable in regression analysis and subsequently dichotomized at 75 years for development of a simplified bedside score. **Results:** Overall in-hospital mortality was 22.3% (37/166). Increasing age (OR 1.08 per year increase, 95% CI 1.04–1.12; *p* < 0.001), septic shock at emergency department admission (OR 7.06, 95% CI 1.29–38.65; *p* = 0.024), and intraoperative vasopressor requirement (OR 6.45, 95% CI 1.34–31.10; *p* = 0.020) were independently associated with mortality. A simplified predictive score based on these variables demonstrated good discrimination, with an area under the receiver operating characteristic curve (AUC) of 0.83. **Conclusions:** Mortality following gastrointestinal perforation was associated primarily with early physiological derangement and patient frailty rather than anatomical or technical surgical factors alone. Early identification of high-risk patients may support perioperative risk stratification and patient-centered emergency surgical decision-making. The proposed predictive score should be considered preliminary and hypothesis-generating, as neither internal nor external validation was performed.

## 1. Introduction

Gastrointestinal perforation represents one of the most severe and time-dependent conditions in emergency surgery and remains associated with high morbidity and mortality despite advances in imaging, perioperative care, intensive care support, and surgical management [1,2,3].

Historically, visceral perforation was considered an absolute indication for emergency laparotomy. However, improvements in diagnostic imaging, broad-spectrum antibiotic therapy, critical care support, and surgical techniques have progressively shifted management toward a more selective and individualized approach [4,5,6,7]. Contrast-enhanced computed tomography (CT) is currently considered the diagnostic gold standard because it allows for accurate assessment of perforation site, extent of contamination, presence of abscesses or free air, and associated ischemic complications [6,7].

Current management strategies are increasingly tailored according to hemodynamic stability, degree of peritoneal contamination, timing of diagnosis, comorbidities, and physiologic reserve. Although minimally invasive, endoscopic, and damage-control approaches may be appropriate in selected patients, prompt surgical source control remains the cornerstone of treatment in most clinically unstable cases [1,2,3,8,9,10].

Despite ongoing improvements in perioperative management, mortality rates remain high, particularly among elderly, septic, and physiologically compromised patients. Within this evolving clinical framework, early identification of prognostic factors is essential to improve risk stratification, optimize perioperative management, and support personalized emergency surgical decision-making.

Existing prognostic tools, such as the Boey and PULP scores, were primarily developed for peptic ulcer perforation and often require multiple variables that may limit bedside applicability in emergency settings [4,11]. Furthermore, these models may be less applicable to heterogeneous gastrointestinal perforations of different etiologies and anatomical locations.

The aim of the present study was therefore to identify early predictors of in-hospital mortality in a consecutive cohort of patients undergoing emergency surgery for gastrointestinal perforation and to develop a simplified clinically applicable risk stratification model based on readily available physiological variables.

## 2. Materials and Methods

### 2.1. Study Design and Setting

This was a single-center retrospective observational study including all consecutive adult patients admitted with gastrointestinal perforation to the General Surgery Unit of the Hospital of Lodi (ASST Lodi) between January 2021 and December 2025.

### 2.2. Study Population

Inclusion criteria were: diagnosis of gastrointestinal perforation; admission through the Emergency Department of the Hospital of Lodi during the study period; or transfer from another hospital with subsequent surgical management at the Hospital of Lodi.

Exclusion criteria were: age < 18 years, appendiceal perforation, iatrogenic perforation, and anastomotic leakage.

The perforation sites considered for the analysis were gastric, duodenal, ileal, colonic, and intraperitoneal rectal perforations.

**Figure 1:**
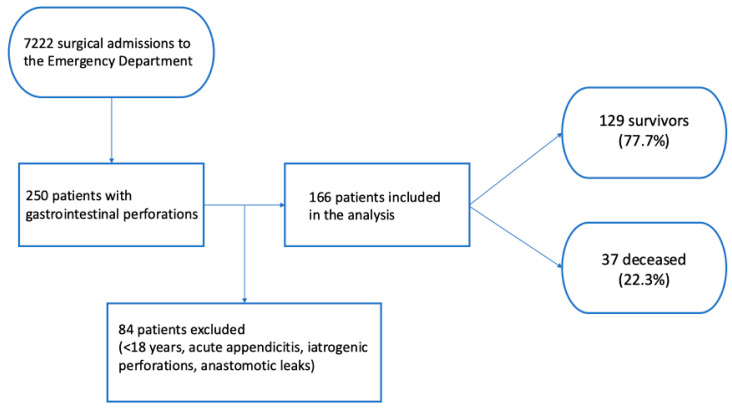
Patients selection.

Eligible patients were identified from the ASST Lodi institutional database. Final selection was performed by reviewing hospital discharge records, including only patients treated at the Hospital of Lodi and excluding those managed at the Hospital of Codogno [Figure 1].

### 2.3. Outcome Definition

The primary outcome was in-hospital mortality. According to this endpoint, patients were divided into two groups: survivors and non-survivors.

### 2.4. Data Collection

Data were extracted from the institutional database using Microsoft Access (Microsoft Corporation, Redmond, WA, USA) and subsequently transferred into Microsoft Excel (version 16.25, 2019; Microsoft Corporation, Redmond, WA, USA).

The following variables were collected: sex, age, year of admission, Charlson Comorbidity Index (CCI), vital signs at Emergency Department admission (heart rate, blood pressure, peripheral oxygen saturation, body temperature, qSOFA score), antibiotic therapy initiated in the Emergency Department, packed red blood cell transfusion in the Emergency Department, laboratory tests at admission (white blood cell count, neutrophil percentage, hemoglobin, platelet count, C-reactive protein, procalcitonin, alanine aminotransferase, creatinine), hypoperfusion and coagulation indices (base excess, lactate, INR), date of surgery, emergency surgery within 24 h, vital signs at anesthesia induction, intraoperative blood transfusion, intraoperative vasopressor requirement, type of anesthesia, site of perforation, surgical procedure, presence of diffuse peritonitis, open abdomen management, anastomosis, surgical approach (laparoscopy, laparotomy, conversion), presence of malignancy, postoperative ward destination, laboratory tests on postoperative day (POD) 1 and POD3, total hospital stay, intensive care unit (ICU) stay, reoperation, Clavien–Dindo classification, and in-hospital outcome.

### 2.5. Missing Data

Missing data were minimal (<5% for all variables). Complete-case analysis was therefore performed, and no imputation methods were applied.

### 2.6. Statistical Analysis

Continuous variables were expressed as median and interquartile range (IQR), whereas categorical variables were reported as counts and percentages. Continuous variables were compared using the Mann–Whitney U test or Wilcoxon test, as appropriate; correlations were assessed using Spearman’s rank correlation coefficient. Categorical variables were compared using the chi-square test or Fisher’s exact test.

Logistic regression analysis was performed to evaluate factors associated with mortality. The following covariates were initially considered: age, sex, CCI, admission vital signs, admission laboratory tests, perforation type and site, surgery within 24 h, vital signs at anesthesia induction, anastomosis, open abdomen, intraoperative vasopressor requirement, postoperative ward destination, reoperation, Clavien–Dindo classification, POD1 and POD3 laboratory tests, total length of stay, and ICU stay.

Only variables with *p* < 0.05 in univariate analysis were considered for multivariable analysis. However, postoperative variables or variables potentially influenced by the outcome were excluded from the final model to reduce the risk of reverse causality, overadjustment, and clinical-statistical collinearity.

All statistical analyses were performed using R 4.5.2 (R Foundation for Statistical Computing, Vienna, Austria) or SPSS for Windows (version 23.0; IBM Corp., Armonk, NY, USA). Model discrimination was assessed using the area under the receiver operating characteristic curve (AUC).

Internal validation techniques such as bootstrapping or cross-validation were not performed because of the limited sample size; therefore, the model should be considered exploratory.

### 2.7. Ethical Approval

The study was conducted in accordance with the Declaration of Helsinki and was approved by the local Institutional Review Board. Owing to the retrospective nature of the study, informed consent was waived.

## 3. Results

### 3.1. Baseline Characteristics

The demographic and clinical characteristics of the study population are summarized in Table 1.

Median age was significantly higher in non-survivors than in survivors [80 years (IQR 13.1) vs. 67 years (IQR 25.7); *p* < 0.001]. Sex distribution was balanced and did not differ significantly between groups.

The colon was the most frequent site of perforation, followed by the ileum and stomach. Septic shock at admission, diffuse peritonitis, need for bowel resection, open abdomen management, intraoperative vasopressor requirement, and intraoperative blood transfusion were more frequent among non-survivors. By contrast, laparoscopic surgery was more frequently performed among survivors.

However, when analyzing mortality in relation to the site, ileal and duodenal perforations show a higher death rate. This finding is likely related to the greater diagnostic difficulty and the rapid septic progression of these conditions.

### 3.2. Univariate Analysis

Univariate analysis identified several variables significantly associated with in-hospital mortality, including age, septic shock at Emergency Department admission, heart rate and blood pressure at anesthesia induction, intraoperative packed red blood cell transfusion, intraoperative vasopressor use, bowel resection, diffuse peritonitis, open abdomen, laparoscopic approach, postoperative C-reactive protein on POD3, major postoperative complications according to Clavien–Dindo classification, reoperation, and ICU stay longer than 48 h.

### 3.3. Multivariable Analysis

To construct a clinically interpretable and methodologically sound predictive model, the selection of variables for multivariable analysis was based not only on statistical significance in univariate analysis, but also on their temporal relationship with the outcome and the risk of clinical or statistical collinearity.

Variables such as ICU stay > 48 h, ICU admission, reoperation, Clavien–Dindo ≥ 3, and total hospital length of stay were excluded from the multivariable model because they were considered to be consequences of postoperative clinical evolution rather than true early predictors of mortality. Their inclusion might have introduced reverse causality and impaired the prognostic value of the model.

In the final multivariable logistic regression analysis, age was independently associated with in-hospital mortality (OR 1.08 per year increase, 95% CI 1.04–1.12, *p* < 0.001). Septic shock at admission (OR 7.06, 95% CI 1.29–38.65, *p* = 0.024) and intraoperative vasopressor requirement (OR 6.45, 95% CI 1.34–31.10, *p* = 0.020) were also identified as independent predictors of mortality [Table 2].

### 3.4. Model Performance

**Figure 2:**
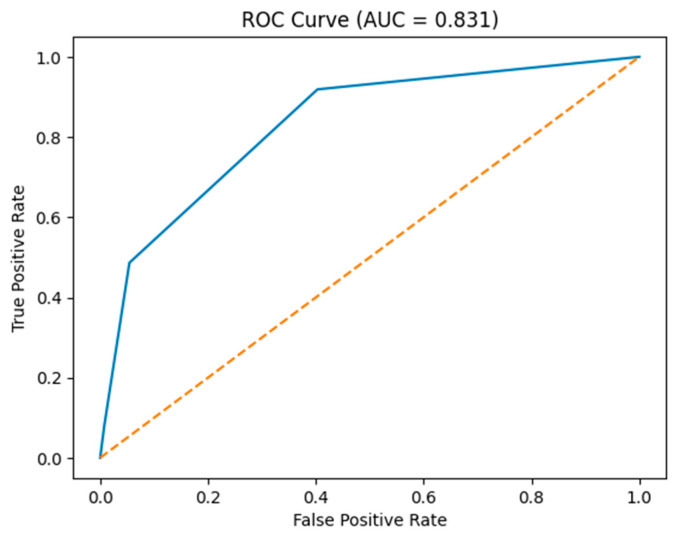
Receiver operating characteristic (ROC) curve of the simplified predictive model for in-hospital mortality in patients undergoing emergency surgery for gastrointestinal perforation. The model demonstrated good discriminative ability, with an area under the curve (AUC) of 0.83.

The predictive model showed good discrimination for in-hospital mortality, with an AUC of 0.83 [Figure 2]. This finding suggests a good ability to distinguish between survivors and non-survivors and supports the potential applicability of the model in emergency surgical settings.

## 4. Clinical Interpretation

### 4.1. Clinical Interpretation of Significant Predictors

Age > 75 years emerged as an independent predictor of mortality. This finding is consistent with previous reports in critically ill surgical patients, in whom advanced age is associated with increased mortality independently of comorbidities and initial disease severity [12,13,14]. Clinically, advanced age likely reflects frailty, reduced physiologic reserve, immunosenescence, and impaired response to surgical and infectious stress.

Septic shock at Emergency Department presentation was also independently associated with mortality and represented one of the strongest prognostic determinants. This is consistent with the literature identifying septic shock as a major predictor of poor outcome in critically ill and emergency surgical patients [15,16,17,18]. In the setting of acute abdominal disease, septic shock at admission indicates advanced disease, persistent tissue hypoperfusion, and limited opportunity for effective preoperative optimization.

Finally, intraoperative vasopressor requirement independently predicted mortality. The need for intraoperative vasopressors is not a direct cause of mortality, but rather a significant marker of hemodynamic instability, reflecting the patient’s inability to maintain adequate organ perfusion during surgical stress [17,18,19].

Overall, the multivariable analysis suggests that mortality in gastrointestinal perforation is mainly driven by patient frailty and early septic-hemodynamic derangement rather than by surgical variables alone.

### 4.2. Predictive Score Development

A simplified clinical score was developed based on the independent predictors identified in multivariable analysis. One point was assigned to each of the following variables: age > 75 years (a clinically simplified version using age > 75 years was derived for bedside applicability), septic shock at admission, and intraoperative vasopressor requirement. The total score ranged from 0 to 3.

Patients were stratified into three risk categories: low risk (score 0), intermediate risk (score 1), and high risk (score ≥ 2). Mortality increased progressively across these categories, supporting the preliminary clinical validity of the model [Table 3].

Mortality increased progressively across risk categories, ranging from 3.8% in the low-risk group to 72.0% in the high-risk group, supporting the potential clinical utility of the simplified score for early bedside risk stratification.

Unlike existing models, the strength of this score lies in its immediate applicability in emergency settings, where rapid decision-making is critical.

### 4.3. Comparison with Existing Scores

Several scoring systems have been proposed for gastrointestinal perforation, including the Boey score and the PULP score. However, many of these models require multiple variables or are difficult to apply in emergency settings. In contrast, the present model is based on only three readily available variables and focuses on early physiologic derangement rather than anatomical factors alone, which may facilitate bedside implementation.

## 5. Discussion

This study systematically evaluated prognostic factors associated with in-hospital mortality in a cohort of patients undergoing emergency surgery for gastrointestinal perforation. Through a stepwise statistical approach including univariate and multivariable logistic regression analyses, increasing age, septic shock at Emergency Department admission, and intraoperative vasopressor requirement emerged as independent predictors of mortality.

These findings suggest that outcomes in gastrointestinal perforation are strongly associated with the patient’s physiological reserve and the severity of septic-hemodynamic derangement during the early phases of care, rather than with surgical or anatomical variables alone [15,16,17,18]. In particular, advanced age likely reflects frailty, reduced physiological reserve, immunosenescence, and impaired tolerance to surgical and infectious stress, all of which have previously been associated with worse outcomes in emergency surgery [12,13,14].

Similarly, septic shock at admission represented one of the strongest predictors of mortality, consistent with the literature identifying septic shock as a major determinant of poor outcome in critically ill and emergency surgical patients [15,16,17,18]. In the setting of gastrointestinal perforation, septic shock likely reflects advanced systemic compromise, persistent tissue hypoperfusion, and reduced opportunity for effective preoperative optimization.

Intraoperative vasopressor requirement was also independently associated with mortality. This variable should not be interpreted as a direct cause of mortality, but rather as a clinically relevant marker of severe hemodynamic instability and impaired physiological compensation during surgical stress [17,18,19]. Overall, the multivariable analysis suggests that mortality in gastrointestinal perforation is mainly associated with patient frailty and early septic-hemodynamic derangement rather than with technical surgical variables alone.

Compared with existing prognostic systems such as the Boey and PULP scores, the present model was designed to prioritize simplicity and bedside applicability in emergency settings. While established scores are valuable, they were primarily developed for peptic ulcer perforation and often require multiple variables that may not be immediately available during emergency surgical evaluation [4,20]. In contrast, the proposed model focuses on three early physiological variables that can be rapidly assessed during initial management and may therefore facilitate bedside implementation in time-sensitive emergency settings.

The simplified predictive score developed in this study demonstrated good discriminative ability (AUC 0.83), suggesting potential utility for early risk stratification. To improve bedside applicability, equal weighting was intentionally assigned to each predictor despite differences in regression coefficient magnitude. Consequently, the proposed score should be considered exploratory and hypothesis-generating rather than a validated clinical prediction tool.

The findings of the present study also highlight the complexity of surgical decision-making in patients with severe physiological derangement and very high operative risk. In selected frail or critically ill patients, aggressive surgical treatment may provide limited expected clinical benefit despite technically adequate source control. Therefore, perioperative decision-making should integrate not only anatomical and technical considerations but also physiological reserve, frailty, expected postoperative recovery, proportionality of care, and patient-centered goals of treatment.

In this context, multidisciplinary discussion and shared decision-making with patients and families remain essential. Therapeutic strategies should aim not only to achieve anatomical correction of the disease but also to ensure appropriateness of care and alignment with the patient’s overall clinical condition and expected postoperative trajectory. Our findings therefore support a shift from a purely disease-centered approach toward a more patient-centered and individualized model of emergency surgical care.

Importantly, the present study should not be interpreted as supporting therapeutic nihilism or withholding indicated surgery. Rather, these findings emphasize the importance of individualized perioperative assessment and careful high-risk operative decision-making in emergency surgical settings.

### Study Limitations

The findings of the present study should be interpreted in light of several limitations. The retrospective single-center design may limit generalizability and introduce the possibility of residual confounding and information bias. In addition, the relatively limited sample size may have increased the risk of model overfitting.

The predictive model should also be considered preliminary, as neither internal nor external validation was performed. Furthermore, the inclusion of heterogeneous perforation etiologies and surgical approaches, together with the exclusion of iatrogenic, anastomotic, and non-operative cases, may have introduced selection bias and limited applicability to broader gastrointestinal perforation populations.

Despite these limitations, the inclusion of consecutive patients and the focus on early clinically available physiological variables may support the practical relevance of the findings in emergency surgical settings.

## 6. Conclusions

In patients undergoing emergency surgery for gastrointestinal perforation, mortality was associated primarily with early physiological compromise and patient frailty rather than with surgical variables alone. Increasing age, septic shock at admission, and intraoperative vasopressor requirement emerged as independent predictors of in-hospital mortality.

The early identification of high-risk patients may improve perioperative risk stratification, facilitate allocation of critical care resources, and support patient-centered emergency surgical decision-making. These findings also emphasize the importance of multidisciplinary perioperative management involving surgeons, anesthesiologists, and intensivists.

The simplified predictive score proposed in this study demonstrated promising discriminative ability and may represent a practical bedside tool for early risk assessment. However, the score should be considered preliminary and hypothesis-generating until validated through internal validation methods and external multicenter studies.

In high-risk patients, emergency surgical decision-making should incorporate not only anatomical disease severity but also frailty, physiological reserve, expected postoperative recovery, and proportionality of care within a shared decision-making framework.

## Figures and Tables

**Table 1:** Demographic table of the study population.

Characteristics	Alive	Deceased	Total Population	*p*-Value
Patients, N	129	37	166	
Age (years), Median (IQR)	67 (25.7)	80 (13.1)	71 (23.5)	<0.001
Sex (male), N (%)	68 (52.7%)	16 (43.2%)	84 (50.6%)	0.354
CCI ≥ 4, N (%)	17 (13.2%)	6 (16.2%)	23 (13.9%)	0.599
Septic shock, N (%)	7 (5.4%)	12 (32.4%)	19 (11.4%)	<0.001
Hyperpyrexia, Median (IQR)	36.6 (1.3)	36.5 (0.8)	36.6 (1.3)	0.88
Antibiotic, N (%)	65 (50.4%)	16 (43.2%)	81 (48.8%)	0.462
MOF, N (%)	3 (2.3%)	1 (2.7%)	4 (2.4%)	1.00
Vasopressors, N (%)	16 (12.4%)	20 (54.1%)	36 (21.7%)	<0.001
PRBC transfusion, N (%)	7 (5.4%)	8 (21.6%)	15 (9.0%)	0.006
Delay, N (%)	7 (5.4%)	3 (8.1%)	10 (6.0%)	0.694
Gastric perforation, N (%)	28 (21.7%)	3 (8.1%)	31 (18.7%)	0.092
Duodenal perforation, N (%)	8 (6.2%)	3 (8.1%)	11 (6.6%)	0.710
Ileal perforation, N (%)	22 (17.1%)	9 (24.3%)	31 (18.7%)	0.342
Colonic perforation, N (%)	64 (49.6%)	19 (51.4%)	83 (50.0%)	1.00
Rectal perforation, N (%)	4 (3.1%)	1 (2.7%)	5 (3.0%)	1.00
Bowel ischemia, N (%)	1 (0.8%)	2 (5.4%)	3 (1.8%)	0.125
Bowel volvulus, N (%)	1 (0.8%)	0 (0%)	1 (0.6%)	1.00
Diffuse peritonitis, N (%)	79 (61.2%)	30 (81.1%)	109 (65.7%)	0.030
Open abdomen, N (%)	4 (3.1%)	10 (27.0%)	14 (8.5%)	<0.001
Surgery with resection, N (%)	87 (67.4%)	32 (86.5%)	119 (71.7%)	0.024
Surgical technique (laparoscopy), N (%)	44 (34.1%)	1 (2.7%)	45 (27.1%)	<0.001
Anastomosis, Median (IQR)	38 (29.7%)	12 (32.4%)	50 (30.3%)	0.839
ICU length of stay (ICU), N (%)	12 (14)	10 (11)	11 (11.8)	<0.001
ICU admission, N (%)	18 (14.0%)	34 (91.9%)	52 (31.3%)	0.023
ICU > 48 h, N (%)	20 (15.5%)	6 (16.2%)	26 (15.7%)	<0.001
Malignancy, N (%)	31 (24.0%)	32 (86.5%)	63 (38.0%)	1.00
Clavien–Dindo ≥ 3, N (%)	13 (10.1%)	12 (32.4%)	25 (15.1%)	<0.001
Reoperation, N (%)	12	13	25	<0.001

**Table 2:** Multivariable analysis.

Variable	OR	95% CI (Lower)	95% CI (Upper)	*p*-Value
Age > 75 years	1.103266	1.042135	1.167983	0.000727
CCI ≥ 4	1.230369	0.270113	5.604343	0.78871
Septic shock	7.061365	1.290082	38.65093	0.024219
Lactate	0.899301	0.571834	1.414295	0.645908
MOF	0.214364	0.000996	46.11535	0.574132
Heart rate	1.018271	0.983	1.054807	0.314104
Systolic blood pressure	1.000657	0.977595	1.024263	0.955975
Vasopressors	6.449895	1.337754	31.09775	0.020205
Diffuse peritonitis	0.657717	0.155368	2.784303	0.569294
Open abdomen	4.128473	0.640919	26.59352	0.135726

**Table 3:** Performance of the simplified predictive score for in-hospital mortality.

Risk Category	Score	Patients (N)	In-Hospital Mortality, N (%)
Low Risk	0	80	3 (3.8%)
Intermediate Risk	1	61	16 (26.2%)
High Risk	≥2	25	18 (72.0%)

Abbreviations: Score variables included age > 75 years, septic shock at Emergency Department admission, and intraoperative vasopressor requirement (1 point each). The predictive model demonstrated good discrimination for in-hospital mortality (AUC 0.83).

## Data Availability

The original contributions presented in this study are included in the article. Further inquiries can be directed to the corresponding author.
